# Application and Development of Fiber Optic Gyroscope Inertial Navigation System in Underground Space

**DOI:** 10.3390/s23125627

**Published:** 2023-06-15

**Authors:** Hang Xu, Lu Wang, Yutong Zu, Wenchao Gou, Yuanbiao Hu

**Affiliations:** 1 Key Laboratory of Deep GeoDrilling Technology, Ministry of Natural Resources, Beijing 100083, China; 2102210033@email.cugb.edu.cn (H.X.); 3002220025@email.cugb.edu.cn (Y.Z.); 2102210032@email.cugb.edu.cn (W.G.); hyb@cugb.edu.cn (Y.H.); 2 School of Engineering and Technology, China University of Geosciences, Beijing 100083, China

**Keywords:** fiber optic gyroscopes, FOG-INS, inclinometer, MWD, pipe-jacking guidance system

## Abstract

Fiber Optic Gyroscope Inertial Navigation System (FOG-INS) is a navigation system using fiber optic gyroscopes and accelerometers, which can offer high-precision position, velocity, and attitude information for carriers. FOG-INS is widely used in aerospace, marine ships, and vehicle navigation. In recent years, it has also played an important role in underground space. For example, in the deep earth, FOG-INS can be used in directional well drilling, which can enhance recovery in resource exploitation. While, in shallow earth, FOG-INS is a high-precision positioning technique that can guide construction in trenchless underground pipelaying. This article extensively reviews the application status and latest progress of FOG-INS in underground space from three aspects—FOG inclinometer, FOG drilling tool attitude measurement while drilling (MWD) unit, and FOG pipe-jacking guidance system. First, measurement principles and product technologies are introduced. Second, the research hot spots are summarized. Finally, the key technical issues and future trends for development are put forward. The findings of this work are useful for further research in the field of FOG-INS in underground space, which not only provides new ideas and directions for scientific research, but also offers guidance for subsequent engineering applications.

## 1. Introduction

The Inertial Navigation System (INS) is a high-autonomy navigation system, which uses gyroscopes and accelerometers as the inertial measurement unit (IMU) to obtain the information of angular velocity and acceleration, and then it is combined with the inertial navigation algorithm to obtain high-precision position, velocity, and attitude information of the carrier [1,2].

According to the type of gyroscopes, INS can be classified into the fiber optical gyro-INS (FOG-INS), the mechanical based gyro-INS (MBG-INS), the ring laser gyro-INS (RLG-INS), the micro electro mechanical system gyro-INS (MEMS gyro-INS), etc. [3]. FOG-INS has outstanding advantages. First of all, compared to MBG-INS, FOG-INS has no mechanical parts, longer service life, and is less susceptible to frictional damage and vibration damage [4]. Secondly, compared to RLG-INS, FOG-INS has a substantial reduction in weight, size, power consumption, and price while maintaining no loss in accuracy [5]. Further, compared with MEMS gyro-INS, FOG-INS has higher accuracy, wider dynamic range, and greater stability, and it is more suitable for complex and variable motion states [6]. However, due to the high price, in the 20th century, FOG-INS was only used in the military fields for precise positioning and navigation, such as missiles, rockets, spaceships, and warships [7]. Later, with the improvement of science and technology, the increase in production capacity, and the decrease in product price, FOG-INS began to be used in civilian fields, such as vehicle navigation, ship orientation, and so on.

In recent years, FOG-INS has also been playing an important role in the field of underground space [8]. For example, in deep earth drilling, with the increasing difficulty of resource exploitation, directional drilling technology is needed to improve recovery. While in urban shallow pipelaying, underground pipelines, subways, and other underground transportation systems are becoming more and more complex, and trenchless technology is needed to reduce surface damage. The precise positioning required by these techniques can be accomplished by FOG-INS [9]. However, it is easy to go up into the sky, and hard to return down to earth. In underground space, FOG-INS faces various challenges, such as narrow space, harsh environment, signal interference, and lack of external information, which can seriously affect the measurement accuracy and stability of IMU, thus reducing the accuracy and reliability of navigation and positioning. However, with the development and the expanded application of FOG technology, FOG-INS has made some progress in the application of underground space.

This paper introduces the application status and latest progress of FOG-INS from three aspects in underground space—borehole trajectory measurement for post-drilling, attitude measurement while drilling (MWD), and trenchless pipe-jacking guidance technology. This paper is structured as follows. Section 2 introduces the working principle of FOG-INS. Section 3 gives the principle of drilling trajectory measurement and the application in post-drilling measurement by FOG inclinometer. Section 4 presents the MWD attitude principle and research status of FOG drilling tool attitude MWD unit. Section 5 describes the product application of FOG pipe-jacking guidance system on in trenchless applications. Section 6 summarizes the key technical challenges faced by FOG-INS in underground space measurement. Section 7 discusses the future development and other application fields of FOG-INS in underground space. Section 8 is the conclusion.

## 2. FOG-INS Working Principle

**Figure 1:**
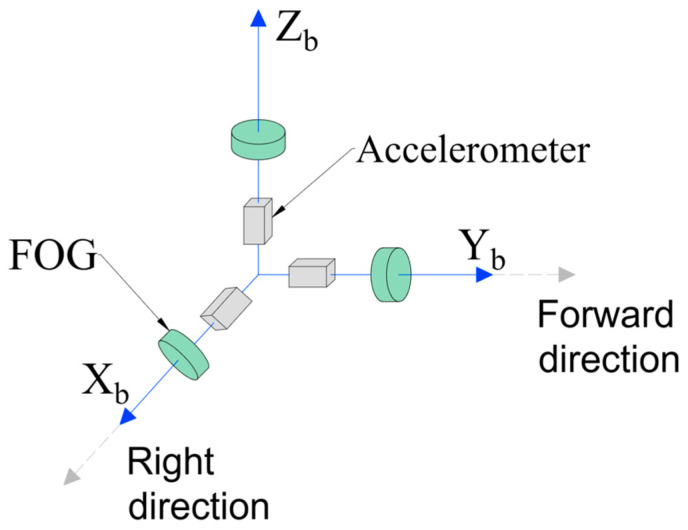
CIMU assembly scheme.

FOG-INS has three FOGs and three accelerometers as inertial sensors, normally, called complete inertial measurement unit (CIMU) [10], which are installed along the *x*-, *y*-, and *z*-axis in the right-hand orthogonal coordinate system. FOGs output angular velocities $\omega_{ib}^{b}={\left[\begin{array}{ccc} \omega_{x} & \omega_{y} & \omega_{z} \end{array}\right]}^{T}$, accelerometers output specific forces $f^{b}={\left[\begin{array}{ccc} f_{x} & f_{y} & f_{z} \end{array}\right]}^{T}$, and the assembly in a carrier is shown in Figure 1.

In this paper, the geographical coordinate system—”East-North-Up”—is selected as the n-frame, and the carrier coordinate system is the b-frame (Figure 1). The transform between b-frame and n-frame came true by the transition matrix $C_{b}^{n}$, which is shown in Equation (1).

(1) $$C_{b}^{n}=\left[\begin{array}{ccc} \cos\psi \cos\varphi +\sin\psi \sin\theta \sin\varphi & \sin\psi \cos\theta & \cos\psi \sin\theta -\sin\psi \sin\theta \cos\varphi \\ -\sin\psi \cos\varphi +\cos\psi \sin\theta \sin\varphi & \cos\psi \cos\theta & -\sin\psi \sin\varphi -\cos\psi \sin\theta \cos\varphi \\ -\cos\theta \sin\varphi & \sin\theta & \cos\theta \cos\varphi \end{array}\right]$$

where; ψ is the azimuth angle, θ is the inclination angle, and φ is the roll angle, which are attitude information.

**Figure 2:**
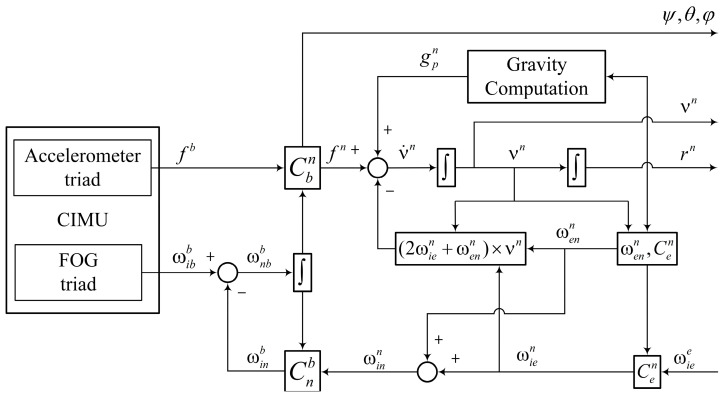
Block diagram of mechanization equation of FOG-INS.

The calculation of FOG-INS is realized through the mechanization equation (Figure 2), in which $C_{b}^{n}$ is used to update the first-order differential equations—${\dot{r}}_{n}$, $\dot{v}$, and ${\dot{C}}_{b}^{n}$. At last, by solving these equations, the real-time position ($r_{n}$), velocity ($v^{n}$), and attitude (ψ, θ, and φ) of the carrier are obtained. The specific meanings of the symbols in Figure 2 are seen in the literature [11].

${\dot{r}}_{n}$, $\dot{v}$, and ${\dot{C}}_{b}^{n}$ consist of a set of first-order differential equations, as shown in Equation (2).

(2) $$\left[\begin{array}{c} {\dot{r}}_{n}\\ {\dot{v}}_{n}\\ {\dot{C}}_{b}^{n} \end{array}\right]=\left[\begin{array}{c} D^{-1}v^{n}\\ C_{b}^{n}f^{b}-\left[2(\omega_{ie}^{n}\times )+(\omega_{en}^{n}\times )\right]v^{n}+g_{p}^{n}\\ C_{b}^{n}\left[\left(\omega_{ib}^{b}\times )-(\omega_{in}^{b}\times \right)\right] \end{array}\right]$$

where; $r_{n}={\left[\begin{array}{ccc} \phi & \lambda & h \end{array}\right]}^{T}$ is the position information, ϕ is the latitude, λ is the longitude, and h is the altitude. $v^{n}={\left[\begin{array}{ccc} v^{E} & v^{N} & v^{U} \end{array}\right]}^{T}$ is the velocity information, $v^{E}$, $v^{N}$, and $v^{U}$ are velocity defined along the East, North, and Up in the n-frame. Additionally, $C_{n}^{b}$ contains attitude information ψ, θ, and φ.

FOG as the uppermost inertial sensor affects the accuracy of FOG-INS, which is based on the constant speed of light and the Sagnac effect. The actual output of the FOG can be expressed in Equation (3) [12]:

(3) $$\tilde{\omega }=K\omega_{ib}^{b}+B_{\omega }+\varepsilon_{\omega }$$

where; $\tilde{\omega }$ is the measurement of the FOG, $\omega_{ib}^{b}$ is the truth-value, $B_{\omega }$ is the constant drift, $\varepsilon_{\omega }$ is the random drift, and K is due to several factors (e.g., the scale factor error).

Through the methods of pre-signal processing, including filtering processing and error compensation, K, $B_{\omega }$, and $\varepsilon_{\omega }$ are eliminated, while the truth-value data $\omega_{ib}^{b}$ is obtained.

From the above formulas and block diagram, it can be seen that the output of the FOG affects the update of the transition matrix $C_{b}^{n}$, which in turn affects the output of the entire FOG-INS. The inertial navigation algorithm uses mathematical integration to solve, and if the errors in Equation (3) are not eliminated, the integration of the error through successive iterations will cause significant interference to the output of FOG-INS.

## 3. FOG Inclinometer

The main application scenario of the inclinometer is to be placed in the borehole after the drilling task is completed, aiming to achieve omnidirectional sensing of the borehole trajectory. In addition, the inclinometer can also be used in the late stage of oilfield development to retest and correct the trajectory of old wells or side drilling windows to explore the reservoir potential, increase the production of oil, and improve recovery [10].

**Figure 3:**
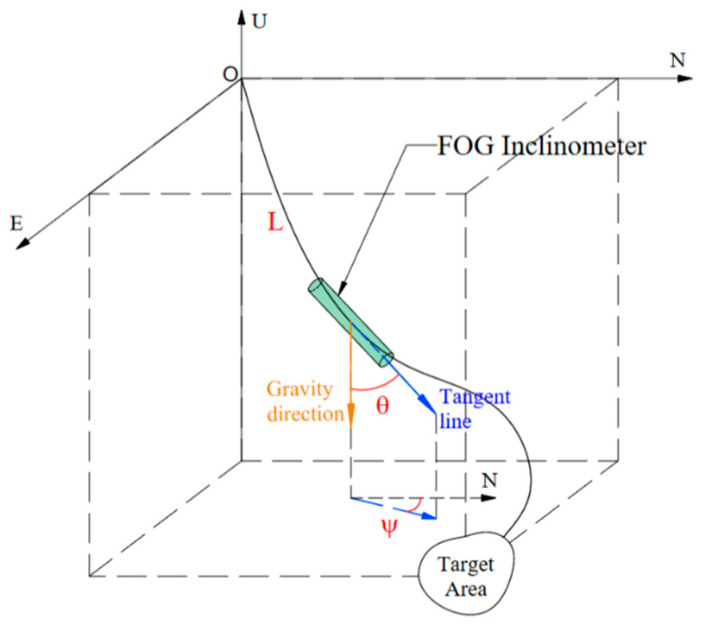
Inclination angle (θ), azimuth angle (ψ), and hole depth (L).

To grasp the borehole trajectory and describe the morphology of the borehole in the subsurface space, the three elements of borehole geometry—hole depth, inclination angle, and azimuth angle—are required [13]. As shown in Figure 3, the hole depth (L) is the distance between the measurement point and the porthole in the borehole, usually measured by cable length. The inclination angle (θ) is the angle between the tangent line of the point where the inclinometer is located and the plumb line. The azimuth angle (ψ) is the angle between the projection of the tangent line of the point where the inclinometer is located on the local horizontal plane and the direction of orientation (usually choose geographic north or magnetic north). Additionally, then, the complete underground borehole curve can be obtained through the mathematical operation of the three-element data of different underground measurements.

The magnetometer inclinometer is a traditional product for inclination measurement, which consists of accelerometers and magnetometers, where accelerometers give the inclination angel, and magnetometers calculate the azimuth angle by measuring the directional difference between the position and the geomagnetic field [14]. However, the magnetometer is susceptible to magnetic field interference from subsurface minerals [15], so external compensation measures are required, such as the use of non-magnetic drill pipes, which not only increases the cost, but also does not completely avoid magnetic interference [11].

FOG-INS has high-precision navigation capability and is less susceptible to magnetic field interference, which can reduce the use of expensive non-magnetic drill collars [9]. In 2000, the University of Calgary modeled the influence of FOG’s resistance to downhole impact force, vibration caused by mud pump noise, bending vibration caused by drill collar rotation, and linear coupling between axial and transverse vibration modes, and it was proved that FOG can resist harsh downhole shock, vibration, and temperature variations. Additionally, it works in conjunction with wireless transmission technologies, such as mud pulse telemetry [16]. In 2005, Ledroz, A.G. et al. [17] discussed the feasibility of using the tactical-grade FOG-IMU LN-2005 as a complete measurement sensor for the downhole inclinometric process, and the simulated downhole experiments demonstrated that the device could resist 40 g of vibration and 75 °C high temperature, proving that the FOG-INS-based FOG inclinometer could be used as an alternative to the magnetometer inclinometer solution.

At present, the typical FOG inclinometer products include the Keeper series from American Scientific Drilling International (SDI), the FIW series from the Institute of Opto-Electronics Technology of Beihang University, China, and the FOG ultra-high-temperature inclinometer from the Institute of Exploration Technology of Chinese Academy of Geological Sciences.

SDI’s Keeper gyro system, whose main performance indexes are shown in Table 1, and the size, weight, length, and adaptability to the environment, such as the ability to resist temperature and pressure, also with the accuracy of measuring the attitude angle of the product, are given special attention, which will determine the range of application of the products. The whole series of products can achieve full attitude measurement, measurement accuracy of ±0.1°, and up to 140 Mpa downhole pressure. At present, there are four generations of products, and different products can be selected for different downhole environments. For example, Wireline Keeper Gyro is adapted to the Φ44.5 mm well, and All Attitude Drop Keeper Gyro is used for high-temperature surveying, extended reach wells, and so on.

**Figure 4:**
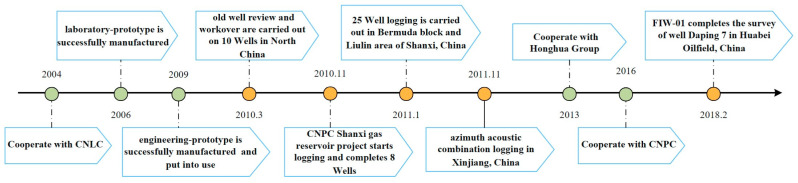
Research and development process and application of FOG inclinometer in Institute of Opto-Electronics Technology of Beihang University, China.

The Institute of Opto-Electronics Technology of Beihang University, China [18,19,20] researched the application of FOG in subsurface logging around the year 2000. The Institute has successively cooperated with China National Logging Corporation (CNLC), China Dongfang Electric Honghua Group Corporation, China National Petroleum Corporation (CNPC), and others. In 2004, the laboratory prototype was successfully developed, and the engineering prototype was put into use in 2009. The development process is shown in Figure 4. It has performed well in the azimuthal acoustic combination logging in Xinjiang of China, the inclinometer operation of well Daping 7 in Huabei Oilfield of China, and so on.

The unit has developed a total of four generations of instrument products. Product performance indexes are shown in Table 2 and Table 3. Each generation of products for different well diameters and more compact structure is shown, of which, the FIW-04 instrument has a weight of 20 kg and a length of 1.2 m.

Aiming at the problems of poor resistance to high temperature and high pressure, poor stability, and large power consumption of inclinometer, the Institute of Exploration Technology of Chinese Academy of Geological Sciences [21] solved the problem of high-temperature and high-pressure multi-point continuous borehole inclination and temperature measurement by designing a FOG ultra-high-temperature borehole inclinometer with a temperature resistance of 270 °C.

In summary, FOG inclinometer research began in 2000, and the market is currently biased toward the application of mature products. It has been in production for many years, it can achieve full attitude measurement, measurement accuracy is accurate to ±0.1°, the minimum is well adapted to Φ44.5 mm, the minimum weight of the instrument is 20 kg, the minimum length to 1.2 m, the temperature resistance is 270 °C, and the pressure resistance is 140 Mpa.

## 4. FOG Drilling Tool Attitude MWD Unit

Although the model of post-drilling measurement is relatively mature and widely used, it still has disadvantages, such as wasting drilling time, the trajectory being easily damaged by drilling fluid, and being unable to detect in real-time [22]. The MWD technology can effectively solve the above problems and can provide real-time and accurate data for drillers to improve drilling efficiency and safety [23]. Therefore, it has gradually developed from post-drilling measurement to MWD.

**Figure 5:**
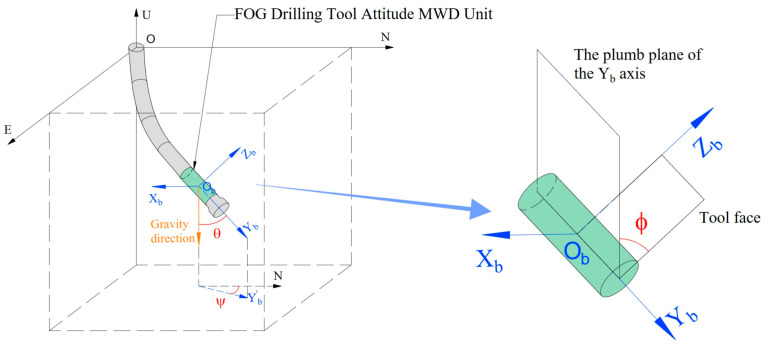
Inclination angle (θ), azimuth angle (ψ), and roll angle (φ).

For the directional drilling industry, MWD instruments are used to measure the drilling tool attitude in real-time, which is the inclination angle, azimuth angle, and roll angle of the bottom hole assembly (BHA). As shown in Figure 5, where the inclination angle and azimuth angle are consistent with those expressed in Section 3 of this paper, the roll angle (φ) is the angle between the positive direction of the carrier vertical axis and the plumb plane where the carrier longitudinal axis is located, which can express the drilling speed of the bit [13].

The FOG drilling tool attitude MWD unit uses FOG-INS technology, which can feedback on the BHA attitude information during drilling for correction and review of drilling. However, FOG-INS faces multiple limitations in MWD. Firstly, IMU must be installed on a carrier for use, and the narrow underground space leads to restrictions on the type of sensors and installation location of IMU, which can affect the measurement accuracy and stability of IMU. Secondly, the harsh environment of high temperature, high pressure, high vibration, and high impact that exists in underground space will reduce the performance and service life of IMU. Thirdly, the presence of signal interference in the underground space, such as geomagnetic fields, can cause errors in the FOG and the accelerometer, which in turn can lead to distortion or drift in the IMU’s output signal. Finally, the lack of external information sources makes FOG-INS unable to navigate directly in combination with other navigation systems, such as GPS and visual radar, etc., thus reducing navigation accuracy and reliability. To solve the above dilemma, researchers have conducted research. This section introduces the current research status of the FOG-based drilling tool attitude MWD unit in three directions: the number of FOG configurations, attitude measurement algorithms, and working environment adaptability enhancement.

### 4.1. Research of the Number of FOG Configurations

In Section 2, CIMU has been introduced, which can realize the full attitude real-time measurement of BHA, and the research technology is relatively mature. The FOG is mounted on the *x*-axis to monitor the change in inclination angle, the *y*-axis to monitor the change in tool face angle, and the *z*-axis to monitor the change in azimuth angle.

However, CIMU has some drawbacks and shortcomings. It is oversized, too high in power consumption, and too high in budget [24]. In the CIMU configuration, FOG is larger, and it has a higher cost and power consumption compared to accelerometers, so reducing FOG’s number of configurations can maximize the requirements and input costs [10], so many scholars have been changing the number of configurations for the FOG to research reduced inertial sensor system (RISS) [25].

**Figure 6:**
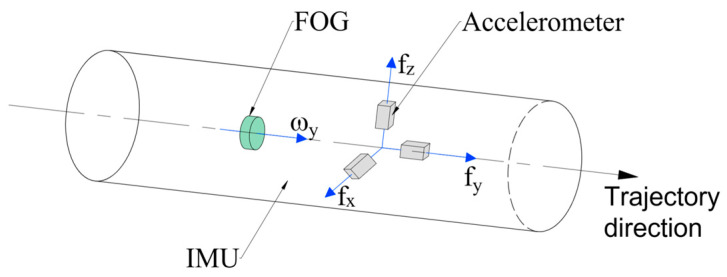
Single-axis FOG-IMU assembly scheme mounted along the *y*-axis.

The literature [10,24,25,26,27,28] proposed a single FOG configuration scheme. FOG is usually installed along the three-axis coordinate system, as shown in Figure 6, for mounting the FOG in the *y*-axis, the advancing direction of the drilling bit, which can improve the measurement accuracy of the tool face angle in near-horizontal drilling and the azimuth angle in near-vertical drilling. Noureldin, A. et al. investigated the effect that could be achieved by mounting a single-axis FOG in different positions, and in 2000 [26], they mounted the FOG on a horizontal surface and simulated the *x*- and *y*-axis angular velocities using the inclination angle and tool face angle output from an accelerometer, which was used for continuous azimuth detection in horizontal wells and high-angle deviated wells, but not for azimuth monitoring when drilling near the vertical. In 2001 [27], they installed the sensitive axis of FOG along the drilling direction, and the azimuth of the near-vertical well was continuously monitored with FOG. However, there exists a 20~45° inclination angle between the wells far from the near-vertical wells and not reaching the high-angle deviated wells, and where neither of the above two methods can achieve accurate attitude measurement, so they proposed a method to select predetermined stations for measurement in this angle range in 2004 [28]. A dual-FOG configuration scheme was proposed in the literature [29,30], and, in [29], they designed an annular FOG parallel to the drilling fluid direction and a second orthogonally positioned FOG in 2004. The annular FOG allows smooth passage of drilling fluid and enhances the measurement accuracy in both near-vertical and near-horizontal sections of the well. Zhang, Y. et al. [28] designed a predigested inertial measurement unit (PIMU) scheme based on rotational modulation in 2012, which calculates the angular velocity of the axis (uninstalled FOG) from the output of the accelerometer, reducing the volume and power consumption compared to the CIMU.

### 4.2. Research of Attitude Measurement Algorithm

It can be seen from Section 2 that the inertial navigation algorithm consists of two parts: pre-signal processing and late navigation calculation process, which all affect the attitude accuracy in MWD. Some studies are as follows.

The purpose of the pre-signal processing is to filter out the excessive offset data in the original output signal of IMU, eliminate the noise and interference of the wrong signals, and avoid affecting the later inertial navigation calculation. Filtering processing and error compensation methods are generally adopted. The literature [12,30,31] used a rotational modulation method to modulate the signal of the FOG, modulating the input signal to a linear sine wave before it is transmitted to the sensor to demodulate the signal and obtain an accurate response of the input. In addition, the common methods of calibration technology, that is, IMU error compensation technology, including multi-position experiments, angular rate experiments, angular vibration experiments, etc., but these all rely on expensive high-precision turntable to achieve, and calibration parameters will deviate as the use environment changes, the current calibration methods for calibration circumstance with small limitations, short calibration time, and multiple calibration targets have become research hot-pots. In 2015, Li, B. et al. [32] selected 12 errors as the main error sources of horizontal drilling and performed five-rotation-in-level-plane (5RILP) in-field fast calibration, and the actual accuracy of the calibrated IMU was improved by 60.6%. In 2020, Cai, X. et al. [33] used the oblique FOG as the measurement in KF to calibrate the MEMS gyroscope error online, and the simulation experiments proved that the calibration method is true and effective. In 2022, Dai, M. et al. [34] measured the rotational angular rate of downhole drilling tools by FOG and used the cosine fitting method to calibrate the deviation of the magnetometer online, and the calibration error was experimentally verified to be less than 5%. In 2022, Lu, J. et al. [35] proposed a four-position and three-rotation (FPTR) calibration sequence using FOG-IMU as a reference information source to calibrate MEMS-IMUs without a turntable and to calibrate large quantities of MEMS-IMUs. In 2023, Bai, X. et al. [36] designed a 30-dimensional KF to calibrate MEMS sensors online using an improved Dijkstra algorithm, and the calibration time was reduced, which provided a new idea for online calibration of FOG.

In the navigation calculation process, due to the use of mathematical integral solutions for navigation, FOG-INS has autonomy, but it also has the disadvantage of error accumulation without introducing external information sources. For this drawback, integrated navigation and Kalman filtering (KF) are always used to correct errors, and two solutions exist. The first one is the correction of the navigation algorithm with the help of external information [37], which can be performed by correction methods, such as coordinate update (CUPT), zero velocity update (ZUPT), and others. ZUPT includes completely stopping the system (similar to station-based surveying drilling) and uses zero velocities as an additional external observation of the KF [14]. In 2022, Ji, S. et al. [38] used the continuous backtracking ZUPT algorithm and improved the azimuthal accuracy by 48.79% compared to the conventional ZUPT. Other constraints can also be introduced. In the literature [10,39], the length of the drill pipe and the speed while drilling are introduced as external observation constraints of KF. In a study by Zhang, C. et al. [39] in 2016, they calculated the drill pipe length by the minimum curvature method (MCM) and set the velocity of the two axes other than the drilling direction as 0, which can effectively suppress the error growth problem of INS with time. Park, B. et al. [40] proposed, in 2018, that taking magnetic field anomalies as constraints and combining magnetic field vectors with data measured by INS could also increase the long-term accuracy of INS.

The second one is integrated navigation measurement with other sensors or other navigation systems to improve the reliability of FOG-INS [13]. For the redundant configuration of MEMS-IMU, the literature [31,33,41,42] proposed a single-axis FOG as a redundant unit to provide additional observation results. The FOG itself as an inertial device is used as a redundant unit, which does not destroy the highly autonomous navigation advantage of INS itself, and it improves the navigation accuracy, but it cannot suppress the accumulation of long-term errors. Zhang, Y. et al. [30] used the position parameters obtained by doing dead-reckoning, based on the length of the drill pipe in 2012, which was used as a measurement of the KF with the INS at the same time, and the semi-physical simulation experiment verified that the positioning error is less than 10 m, which can improve the shortcomings of the INS alone.

Combined with other navigation systems, it can also suppress the long-term divergence of errors. In 2012, Wang, X. et al. [43] designed a magnetic inertial altitude-heading reference system (AHRS), consisting of three-axis magnetometers, accelerometers, gyroscopes, and a FOG plus. They measure the three-dimensional trajectory of urban underground pipelines within 200 m in complicated field conditions, where electromagnetic disturbances exist, and the maximum horizontal error is less than 0.4 m. In 2022, Dai, M. et al. [34] designed a small-volume combined magnetic heading measurement scheme of vertical single-axis FOG, tri-axial MEMS accelerometer, and tri-axial magnetometer, which can improve the accuracy of magnetic heading angle measurement in the case of continuous rotation of a large angle. In addition, the combination of GPS and FOG-INS is widely used in many fields because it can provide accurate initial position, but the inability to accurately identify satellite signals in the underground space will lead to information retention or even reception failure, and this phenomenon is more obvious as the drilling depth increases [44,45]. Guan, L. et al. [46] proposed that GPS signals can be used at two ends of underground pipeline measurements. Laguillo, M. et al. [47] proposed to enhance the signal reception frequency in shallow horizontal drilling, and they also used GPS signals in the intermediate stations of pipe-jacking guidance. All of the above methods have been experimentally verified to improve the signal reception problem of GPS in underground space.

### 4.3. Research of Working Environment Adaptability Improvement

In response to the harsh conditions of narrow space, high temperature, and high vibration downhole, some scholars have made the following studies.

(1) Research on FOG-INS miniaturization

Zhang, C. et al. [48] proposed to increase the fiber length by changing the shape of the fiber coil to reduce the size while ensuring the accuracy of the FOG. Noureldin, A. et al. [27] designed a torus FOG that facilitates drilling fluid circulation by changing the FOG fiber to an elliptical torus winding in response to the strict space constraints of MWD instruments downhole. In addition, the use of a three-axis integrated design reduces the volume and power consumption of the IMU by sharing a common light source for the three-axis FOG.

(2) Research on vibration resistance and temperature resistance

The effect of interference signals on FOG-INS can be seen in Section 2 and Section 4.2. This effect is more pronounced in harsh underground environments, especially under high temperatures and high vibrations. The error model identification and parameter determination method, namely, the modeling method, has auto-correlation, power spectrum density (PSD), Allan variance analysis, and so on. Then, the errors are eliminated by a number of methods, such as sensor calibration, filtering methods, data fusion, etc.

In the research on vibration resistance, in general, in IMU, accelerometers are more disturbed and affected by vibrations, and Yue, T. et al. [49] proposed to use random vibration generated by the linear vibration table to calibrate accelerometers to compensate for vibration disturbances. Wang, L. et al. studied the vibration of the gyroscope and concluded that the linear vibration will generate the angular vibration at the same frequency as a way to disturb the stability of the gyroscope in 2018 [50]. To mitigate the vibration, they used a 36-order Extended Kalman Filter (EKF) to identify the coefficients in the error model in 2021 [51]. In addition to KF, which is commonly used in INS, other filtering methods are also used in the anti-vibration study of FOG-INS. In 1989, Rehm, W.A. et al. [52] selected the method of the adaptive filter to remove vibration noise and the addition of seismic protection shell, which can withstand 40 g of vibration. In 2018, Ren, C. et al. [53] used a wavelet filtering method to suppress the interference of downhole impact and designed a combined measurement scheme of single-axis FOG and dual-axis MEMS accelerometer, and the system could withstand more than 100 g impact under laboratory conditions.

In the research on temperature resistance, the temperature resistance of FOG is related to the internal optical fiber torus of FOG, which is not easily corrected by algorithms and filtering after manufacturing, but FOG is more resistant to steady-state temperature than changing temperature, and in the actual drilling, the drilling fluid will reduce the temperature gradient, and it will also mitigate the influence of sudden temperature change, and it is usually more cost-effective to be equipped with the insulation bottle for use. So, the thermal error compensation method is usually used. In 2004, Mohr, F. et al. [54] determined the composition of thermally induced bias error of a fiber optic gyroscope and designed a series of temperature experiments to verify the adequacy of the chosen modeling items. In 2012, Zhang, Y. et al. [55] focused on the dynamic angular velocity modeling and error compensation of a single FOG over the whole temperature range and proposed a thermal error compensation method based on an orthogonal design. In 2019, Liu, Y. et al. [56] proposed a FOG thermal error compensation scheme using Shupe error and improved the accuracy of thermal error compensation by using the finite-element method (FEM) and uniform mixed-data design method.

In summary, as MWD technology and FOG-INS technology have matured in recent years, more and more research has been conducted for them. This section reviews the research on FOG-based MWD units for drilling tool attitude, and most of the current research is focused on sensor calibration, integrated navigation information fusion, and enhancement of environmental adaptability.

## 5. FOG Pipe-Jacking Guidance System

Trenchless technology is an engineering method of excavation construction based on not carrying out ground “open excavation”, including numerous construction methods [57]. Among them, pipe-jacking technology is a pipeline and tunneling construction method for segment laying that uses the main jacking cylinder to jack the pipe-jacking machine and pipe segment from the working well to the reception well and uses the cutting head of the pipe-jacking machine to rotate forward to cut soil and rock, and it can cross complex geological conditions, such as lakes, mountains, and swamps [58].

Pipe-jacking construction requires high accuracy, and the deviation of the pipe axis needs to be less than 50 mm, and, to lay the pipe along the preset axis, it needs to be guided during pipe-jacking [59,60]. At present, the commonly used pipe-jacking guidance methods include the laser target method and the total station guidance method. The laser target method is mostly used for short-distance straight pipe-jacking because the laser can only propagate along the straight line, and the method is not suitable for curved pipe-jacking. Moreover, with the increase in pipelaying distance, the spot of the laser beam on the target will diverge and the measurement error will increase, increasing the construction error of long-distance pipe-jacking. The total station requires internal pipe-jacking with pass-through conditions and simultaneous observation of three target prisms, which is generally used in pipe-jacking projects with pipe diameters greater than 1.6 m. For medium and long-distance pipe-jacking projects, professional personnel are required to enter the pipeline, and multiple additional total stations are needed to meet the measurement requirements, which leads to the limitations of repeated installation and unfavorable observation conditions in pipe-jacking projects.

**Figure 7:**
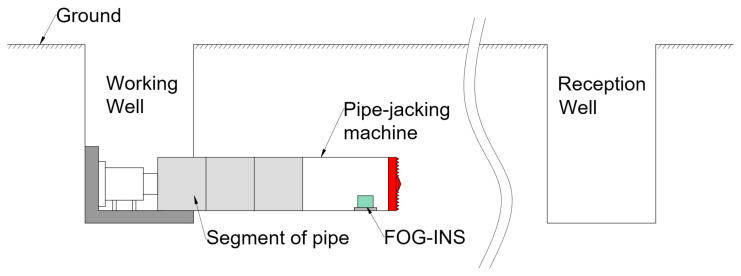
FOG pipe-jacking guidance system.

For pipe-jacking construction, FOG-INS technology can provide real-time attitude and position information to the pipe-jacking machine based on its autonomous navigation advantages, without the need to install and measure the coordinates of external prisms and without the requirement of pass-through condition, and the equipment can be installed once to complete the entire pipe laying [61], and the FOG pipe-jacking guidance system is shown in Figure 7.

Some companies have already applied FOG-INS to pipe-jacking guidance. Typical products available now are the TUnIS Navigation MT gyroscope system from Bruchsal, Germany VMT Co., Ltd., the TMG series FOG-INS and PN series guidance system from Tokyo, Japan TOKYO KEIKI Co., Ltd., and the curved pipe-jacking automatic guidance system from Shanghai, China Lixin Measurement Co., Ltd. The measurement principle of the gyroscope guidance system from VMT is based on the gyroscope and dead reckoning with millimeter-level accuracy.

The PN-200 system from TOKYO KEIKI introduces the odometer based on the FOG. The heading information of the pipe-jacking machine is determined by the north-pointing function of the FOG, and the guidance measurement of the pipe-jacking machine can be realized by combining the measurement results of the odometer. At present, the main products of the company are TMG-32F and PN-S1 guidance systems. The product performance parameters of TMG-32F are shown in Table 4. Due to the demand of pipe-jacking conditions, the measurement range of the inclination angle does not need to reach the standard of the inclinometer.

The curved pipe-jacking automatic guidance system from China Lixin Measurement Co., Ltd. can meet the minimum diameter of a 500 mm pipe-jacking machine and can be configured with different ranges of pressure sensors to adapt to the height difference of 100 m tunnel, and the error can be corrected at least once every 50~100 m. The product performance parameters are shown in Table 5.

In summary, in the trenchless pipe-jacking guidance system, since the measurement methods, such as total station and laser target are no longer applicable in the applicable scenarios of small diameter, large elevation, long distance, and large curvature, FOG-INS technology can be a perfect substitute, which can provide high accuracy, high stability, and high reliability of guidance information in pipe-jacking construction.

## 6. Key Technical Issues

### 6.1. Miniaturization of FOG

The miniaturization research of FOG aims to adapt it to the narrow underground working space. Since the borehole diameter in the underground space is generally 100~315 mm and FOG needs to be equipped with an insulation bottle, a FOG inclinometer is usually designed to be long and cylindrical and less than 80 mm in diameter, while short sections for attitude measurement in MWD require smaller sizes.

To achieve the miniaturization of FOG, the research is mainly divided into two directions. The first method is to reduce the size of the FOG itself, either by changing the structure of the internal optical fiber, such as winding the elliptical torus and other special fiber winding methods, or by conducting research and development work on special optical fibers that improve the fiber material, structure, and process. Multi-axis FOG can also share an SLD light source for structural integration. The second method is to reduce the number of FOG devices, according to the difference in attitude angle accuracy required by the actual working conditions and to conduct research on reduced inertial sensor system (RISS) for single-axis, dual-axis, and oblique FOGs, which can vary the number of FOG units, depending on the application scenario. For example, in the pipe-jacking field, the demand for inclination angle is reduced, so the dual-axis FOG selection can be chosen.

### 6.2. Integrated Navigation Technology Based on FOG

In the FOG-INS measurement process, the use of mathematical integral solutions for navigation often results in the long-term accumulation of small errors, which can be eliminated by using the integrated navigation method to improve navigation accuracy and robustness.

There are two research directions for FOG-based integrated navigation techniques. The first one is to introduce external information quantity, which can be used by ZUPT, CUPT, etc. Drilling speed information, drill pipe length information, and the geomagnetic signal can also be fused into the FOG-INS calculation as constraints. The second is a combination with other sensors and navigation systems to achieve data fusion and complementarity. Based on FOG-CIMU, other inertial devices can be introduced to be a redundant configuration, for example, magnetometers can supplement magnetic information, odometers can supplement elevation information, etc. In addition, other navigation methods could be combined with FOG-INS, such as GPS, dead-reckoning, magnetic beacon technology, and visual radar.

It is also possible to perform composite measurements, such as inclinometers and MWD technology used in conjunction. The current operation mode to prevent oilfield borehole collision in oil drilling is to use an inclinometer for surface orientation in advance. MWD is used to measure borehole trajectory in real-time during drilling, and then the inclinometer is used again to measure the casing trajectory after the completion of drilling. This composite measurement method is the optimal solution in the case of harsh downhole environments, such as high vibration, excessive drilling fluid interference, etc. when MWD cannot reach the optimal use conditions.

### 6.3. The Anti-Interference Ability of FOG-INS in Harsh Environments

In the harsh environment of high vibration, high pressure, and high temperature in the underground space, there are several solutions to enhance the immunity of FOG-INS in harsh environments.

First of all, the anti-vibration of the drilling tool attitude measurement unit is put forward in MWD with high standards, which is required to bear at least 30 g of vibration and must be retrofitted with anti-vibration devices, and vibration signal filtering, such as KF, EKF, and wavelet filter, which can be used to mitigate the interference of vibration. In oil wells, the inclinometer is generally subjected to the vibration of no more than 2 g and impact of no more than 10 g, both of which are far below the high vibration and large impact environment in the aviation industry, so there will be no major problems in use.

Secondly, at the bottom of well several kilometers deep, the measurement unit needs to withstand pressures of tens or even more than a hundred megapascals, so it is necessary to consider the pressure protection of the measuring device and the sealing problem of the instrument connection.

Finally, the temperature gradient in the formation is about 3 °C/100 m, so the downhole temperature at 3000~5000 m is about 90~150 °C. In general areas, when the drilling depth reaches 8000 m, the temperature will reach 250 °C, and the temperature of ultra-deep wells will be higher, so it is necessary to install external high-efficiency insulation bottles and temperature detection unit, and, in addition to this, the measure of thermal compensation can also be selected. To avoid high-temperature damage inside the system, the measurement time should be compressed as much as possible, for example, by using an automatic intermittent power supply.

## 7. Discussion

In underground space, FOG-INS faces several challenges, while also providing tremendous research spaces. How FOG-INS adapts to these challenges gives full play to its advantages, and it solves the key technical issues raised in Section 6, which are the next research directions. If the above requirements are met, FOG-INS will have a wider application of underground space in the future.

(1) It can be used in the exploitation of hot dry rock. Hot dry rock is one of the renewable geothermal energies, which is stored at depths of more than 3000 m in crevices of stone, the ambient temperature around which can be over 200 °C. Therefore, the precision and temperature requirements of directional drilling are very high. However, at present, limited by drilling technology and equipment, the utilization of hot dry rock accounts for only a small fraction of the proven reserves. If FOG-INS can withstand high temperatures, it will be of great value in seeking and mining for hot dry rock.

(2) It can be used in near-bit MWD. Currently attitude measurement can only be achieved around 10 m away from the bit. If miniaturization and resistance to vibration interference of FOG-INS during drilling can be realized, FOG-INS are integrated closer to the drill bit. In this way, the attitude angle obtained is the attitude angle of the bit, which can better feedback drilling information in real-time, and the real sense of near-bit measurement will be realized.

## 8. Conclusions

This paper studies the current application of FOG-INS in the underground space and innovatively reviews the development process and product technologies from three fields, including FOG inclinometer for the borehole trajectory measurement of post-drilling, FOG drilling tool attitude MWD unit for MWD, and FOG pipe-jacking guidance system for trenchless pipe-jacking. According to this study, the key technical problems are analyzed, and the future development trends are looked forward to. It is of great significance to understand the current research status and indicate future research directions. First, the principle of borehole trajectory measurement, based on FOG, is proposed, and FOG inclinometer products of three organizations are introduced. The post-drilling measurement can rule out the interference from factors, such as drilling tool vibration, but due to the erosion of drilling fluid and the long construction period required, MWD has become a mainstream study. Second, the research of the FOG drilling tool attitude MWD unit is presented from three aspects: the configuration number of FOG, the attitude measurement algorithms, and the improvement of working environment adaptability. The current research focuses on the directions of online sensor calibration, integrated navigation information fusion, and the improvement of environmental adaptability. After that, two products applied in the pipe-jacking guidance system are introduced, FOG-INS can make trenchless pipe-jacking fit the working environment of small diameter, large elevation, long distance, and large curvature. At least, the key technical issues and the trends for the future application of FOG-INS in underground space are put forward. The current research status shows that FOG-INS has great research value in the field of underground space. FOG-INS will continue to expand the scope of research in the future and develop techniques of optimizations, miniaturization, simplified and precise navigation, environmental adaptability improvement, and integrated navigation.

## Figures and Tables

**Table 1:** Main performance indexes of the Keeper series.

Product Mode	Outside Diameter /(mm)	Weight /(kg)	Length/(m)	Temperature Resistance /(°C)	Pressure Resistance/ (MPa)
Wireline Keeper Gyro	44.5 (1.75″)	50	5.5	149	140
Memory Keeper Gyro	47.0 (1.85″)	54	5.8	204	
All Attitude Drop Keeper Gyro	54.0 (2.125″)	63			
Sightline Keeper Gyro	76.2 (3.00″)	227	8.5		

**Table 2:** Main performance indexes of the FIW series.

Product Mode	Outside Diameter /(mm)	Weight /(kg)	Length/(m)	Temperature Resistance /(°C)	Pressure Resistance/ (MPa)
FIW-01	98	67	2.56	−25~175	140
FIW-02	76	60	2.8		
FIW-03	48	45	1.7	25~85	
FIW-04	45	20	1.2	0~85	

**Table 3:** Range and accuracy of angle measurement of the FIW series.

Product Mode	Inclination Angle		Azimuth Angle	
	Range/(°)	Accuracy/(°)	Range/(°)	Accuracy/(°)
FIW-01	−90~90	±0.1	0~360	±0.1
FIW-02	−90~90	±0.2		
FIW-03	−90~90	±0.2		±2
FIW-04	−70~70	±0.2		

**Table 4:** Measurement accuracy of TMG-32F of TOKYO KEIKI.

Service Ambient Temperature/(°C)	Measuring Item	Measuring Range/(°)	Accuracy (°)
−25~+55	azimuth angle	0~360	±0.05° s ϕ
	pitch angle	−15~+15	±0.3° s ϕ
	roll angle	−15~+15	±0.3° s ϕ

**Table 5:** Measurement accuracy of the curved pipe-jacking automatic guidance system of China Lixin Measurement.

Service Ambient Temperature/(°C)	Measuring Item	Measuring Range/(°)	Accuracy (°)
−10~+60	azimuth angle	0~360	±0.05°
	pitch angle	−30~+30	±0.01°
	roll angle	−30~+30	±0.01°

## Data Availability

This article is a review and does not involve its own data. All the data involved in the article are quoted from references.
